# Section of the Symphysis Pubis

**Published:** 1810-06

**Authors:** J. S. Blount

**Affiliations:** Member of the Royal College of Surgeons, London. Paris


### To Dr. BRADLEY.

Dear Sir,

If the following observations, extracted from the medical periodical publications of this capital, and of Montpellier, can, in the slightest degree, contribute to the utility of your interesting Journal, I beg you will accept them as a mark of the sincere respect and esteem, with which I have the honour of being,

Yours, &c.
J. S. BLOUNT,
Member of the Royal College of Surgeons, London.

*Paris, February* 20, 1810.

*Journal*

---

*Journal de Medicine de Mess. Corvisart, Leroux, Boyer, &c. January* 1810.

M. SAVARESSI, in his account of the medical history of the army of Naples, to which he was chief physician, observes, that during the campaign of Calabria, autumn 1806, emetics were generally hurtful in the periodical remittent fevers, and sometimes struck the patient as with sudden death. They were useful only in those cases of intermittent fever called *local* or *irritative*, a species recognized by Joseph Frank, of Vienna, and by Rubbini, of Parma. From October to December, a *diarrhœa* with *lienteria*, accompanied with emaciation and great prostration of strength, occurred to most of those who were convalescent of intermittent fever, and particularly to horsemen; it was most commonly mortal, with marasmus; bitters, tonics, excitants, glysters of g. arab. wine, bark, turpentine, bals. capivi, and friction, especially on the spine of the back, saved a few. In those who died, the lungs and abdominal viscera were found to be seriously affected, though no external symptom gave reason to suspect it. Some quartan autumnal fevers were cured by the reiterated application of blisters to the nape of the neck and between the shoulders; others by the use of gall nuts alone, or combined with *mur.* of *amm.* with calomel, or with black oxyd of iron; others by opium, given to the dose of sixteen grains daily. Dr. Picart, employed at the Hospital of Granili for the treatment of psoric patients, has observed several obstinate quartan intermittents disappear on the eruption of the itch; he also had an opportunity of observing a case of excessive head-ache, which no means whatever could alleviate; it occasioned death by the violence of the pain. On examination, the brain was found filled with hydatids.

Dr. Gaetano Monti, physician to the civil establishment in the island of Ischia, has observed that the warm baths of this island have the property of curing the largest and most inveterate exostosis from a venereal cause, provided the patient has previously undergone a mercurial treatment. It is the intention of Dr. Monti to publish some account of these waters, and their efficacy in certain diseases.

Mr. Tartas, assistant surgeon of the 15th regiment of French dragoons, gives the following case of a leech situated in the back of the throat, behind the velum pendulum palati. A soldier complained of head-ach and difficult respiration, sometimes even to suffocation; an evacuation of a small quantity of black blood from the mouth and nose then occurred, followed by a remission of all these symptoms; no fever, no fixed pain in the throat, nor any thing to be seen on examining the fauces. These symptoms, with an evacuation of blood, occurred every two hours, nor did any of the means made use of, as baths, astringent gargles, sedatives, antiphlogistic diet, &c. have any effect. The patient became daily more and more debilitated by the frequency of the hæmorrhage, loss of appetite, and loss of sleep, when, on the 16th day of these accidents, a blackish body was seen at th . back part of the throat, and recognised to be a leech; an instrument was immediately sent for to extract it, but in the interval it emptied itself, and withdrew up behind the velum pendulum palati; the patient voiding as before, black blood by the nose and mouth. The throat was then occasionally examined till the animal having filled itself, was again seen to descend behind the uvula; it was then seized hold of with a pair of forceps in the left hand, and torn from its bite with the finger and thumb of the right; the patient had no return of any of the symptoms. This accident has occurred several times in Spain, where the soldiers, obliged to satisfy their thirst by stooping down and drinking with their mouths in filthy stagnant water, are liable to suck in with it these animals when very small, and which, fixing themselves to some part of the throat, may there increase in growth, and occasion symptoms similar to those above mentioned.

*Section of the Symphysis Pubis.* At the end of the first pregnancy, the short diameter of the brim of the pelvis was found to be rather more than 2 ½ inches; the child being dead, the head was opened and extracted. At the end of the second pregnancy, a strong healthy leg came down; section of the symphysis proposed and put in execution. The separation of the pubis being ¾ of an inch, the extraction was attempted by the feet; separation gradually increased to 1 ¼ inch; *arms* and *shoulders* then brought down; pulsation of the umbilical cord felt; great difficulty in extracting the head; forceps used; their appli cation took up some time; separation of the pubis increased to 2 inches; head brought out with the forceps; child dead. The pubis remained slightly separated for some days after the operation; the left side projected also more than the right; this was effaced in a few days. A tight broad broad bandage was worn round the pelvis; patient got up on the 6th day; no pain after the 12th. After the 18th, went up and down one pair of stairs daily; pain in the left groin, which was dissipated by some slight application to the part; the wound was healed in about three weeks, and in about thirty days, she left off the circular bandage round the pelvis.

---

The Journal of the Medical Society of Paris, for Jan. 1810, published by Dr. Sedillot, contains, 1st. the eighteenth article, on Semiology, by M. Double, M. D. containing semiological considerations on the signs deduced from the examination of the abdominal region.

2dly. Observations on the abuse of purges, by Mr. Rechou, who gives the case of a man who was reduced almost to a state of marasmus by fifteen purges administered for an ulcer of the leg. He observes, that in dropsy, after the third or fourth drastic purge, the belly swells, and the excretions of this cavity are suppressed; the excretory vessels being irritated, their membranes inflamed, and sufficiently swelled to close up the mouths of those vessels. Mr. Rechou remarked also, that in an epidemic putrid fever, all those patients recovered, in whom the application of a blister on the thigh produced a superficial gangrene on the part where it was applied.

3dly. History of the Medical Constitution of Paris for the last six months of the year 1809, by Dr. Double. He observes, that the application of an ounce of salt, powdered and mixed with the yolk of an egg, to an anthrax, frequently arrests the progress of the disease, and facilitates the separation of the escar. He mentions a case of a small cancerous ulcer which had existed for seven or eight years, at the corner of the mouth, spreading over one side of the face, from the eye to the neck, in the course of one night, and the patient died at eight in the morning; a young cat, allowed to approach this dreadful ulcer, died in a few minutes after.

4thly. A review of books.

---

Annales Chimiques de Montpellier, December 1809, formerly published under the title of Annales de la Societe de Medicine de Montpellier. 1st. 5th. and last paper on researches into the phænomena which announce, or which prove, malignity in acute diseases, by Dr. Baumes, Professor of Medicine at Montpellier.

2d. A case of constipation of the bowels, from a separation ration of the recti muscles to the distance of three inches; the four fingers could be placed between them. The patient only went to stool once every six days; the evacuations were bloody, accompanied with excessive pain and copious eructations; a bandage round the abdomen effected a cure.

3d. The white oxidule of Bismuth is advised as a remedy in cases of cramps or spasmodic affection of the stomach, from a quarter of a grain to two grains once or more times a day. The following preparation is also recommended as a specific for the same complaint; Corall. cors. ʒv. Aloes socot. gr. iiij, a pinch of wormwood leaves, boil in four ounces of water; let it infuse for ten minutes, strain and take the whole at the beginning of the attack, when the stomach is empty.

4th. Four ounces of a saturated infusion of the dry leaves of the Lamium Album, taken three times a day has been given with success in cases of *fluor albus*; its use must be continued for three or four weeks.

5th. Four cases of dropsy *(ascites)* out of five were cured by a vapour bath. Boiling water in a tub; eight or ten handfuls of ants, with the sand in which they are found thrown into it; the patient is then placed over the tub, and so covered, that the whole of the body, except the head, is exposed to the vapour of the bath; perspiration, and a copious flow of urine are the result.

6th. Dr. Weber attributes to the antiphlogistic means used after the operation for inguinal hernia, the frequent non-success of this operation. In those cases where no accident complicated the disease, he recommends a stimulating comforting treatment. Of six patients operated on by him, and so treated, five were cured

7th. *Iritis,* inflammation of part of the eye, with pain and epiphora seen to occur frequently after the operation for cataract, has been successfuly treated by hyoscyamus, both internally and externally, one grain given occasionally, and a few drops of a solution of one drachm in an ounce of water, introduced between the eye-lids every four hours.

8th. Itching of the parts of generation and anus, successfully treated by washing the parts with a solution of sublimate.

9th. Dr. Ritter gives a case of those cured by injecting an alkaline solution into the bladder, and by drinking the same preparation. These last five articles are given as copied from some foreign Journal, which is not named.

